# Applications of biomass probe in PAT

**DOI:** 10.1186/1753-6561-7-S6-P89

**Published:** 2013-12-04

**Authors:** Chandrashekhar K Nanjegowda, Nirmala K Ramappa, Pradeep V Ravichandran, Deepak Vengovan, Saravanan Desan, Dinesh Baskar, Ankur Bhatnagar, Anuj Goel

**Affiliations:** 1 Cell Culture Lab, Biocon Research Limited, Bangalore, India

## Introduction

In biologics manufacturing, process consistency is essential to produce the desired product quality over the product life cycle. Process monitoring is an important tool to achieve consistency and robustness. Typical process parameters monitored at upstream are viable cell concentration (VCC), viability, titer, nutrient levels, waste metabolites, osmolality, pH, DO and pCO_2_. Traditionally pH, DO and pCO_2 _are monitored using online sensors while others are measured by offline sampling methods. With recent advances in sensor technology, probes are now available to reliably estimate some of these parameters online. One such tool is biomass probe which estimates VCC by measuring capacitance in the bioreactor. In this work two cases are presented where biomass probe has advantages over traditional offline sampling and can be used as an effective PAT tool to monitor and improve process consistency and robustness.

## Experimental Approach

CHO and NS0 cell lines were used to run fed batch (70L) and perfusion (1KL) runs. The perfusion bioreactor used two Spin filters (SF) as cell retention device that could be switched when required. Biomass probe readings were compared to the VCC estimated by offline sampling.

## Results and discussions

In Fed Batch runs, offline and online VCC values were very comparable during the initial days of the run and deviated with increased process duration and drop in cell viability. In the Perfusion Batch, the offline and online VCC values were comparable throughout the run.

The current work focusses on the phases where online biomass probe can be reliably used to improve efficiencies of both Fed Batch and Perfusion processes.

### Case 1: Improving process efficiency in Fed batch

#### Inoculum propagation and transfer

Inoculum plays a critical role in the process performance; therefore inoculum consistency is very important. Inoculum development step requires cells to be transferred to the next stage while they are in the exponential phase. This is normally done by sampling the seed bioreactors, measuring the cell counts and transferring cells to the next stage.

As this requires sampling for cell counting, due to rapid cell growth in this phase, generally a wide range of acceptable cell concentration is given for practical reasons. Although during this broad range of acceptable cell concentration, cells are in their exponential phase, the volume of inoculum added into the bioreactor changes the spent media ratio inside the production bioreactor considerably.

**Figure 1:**
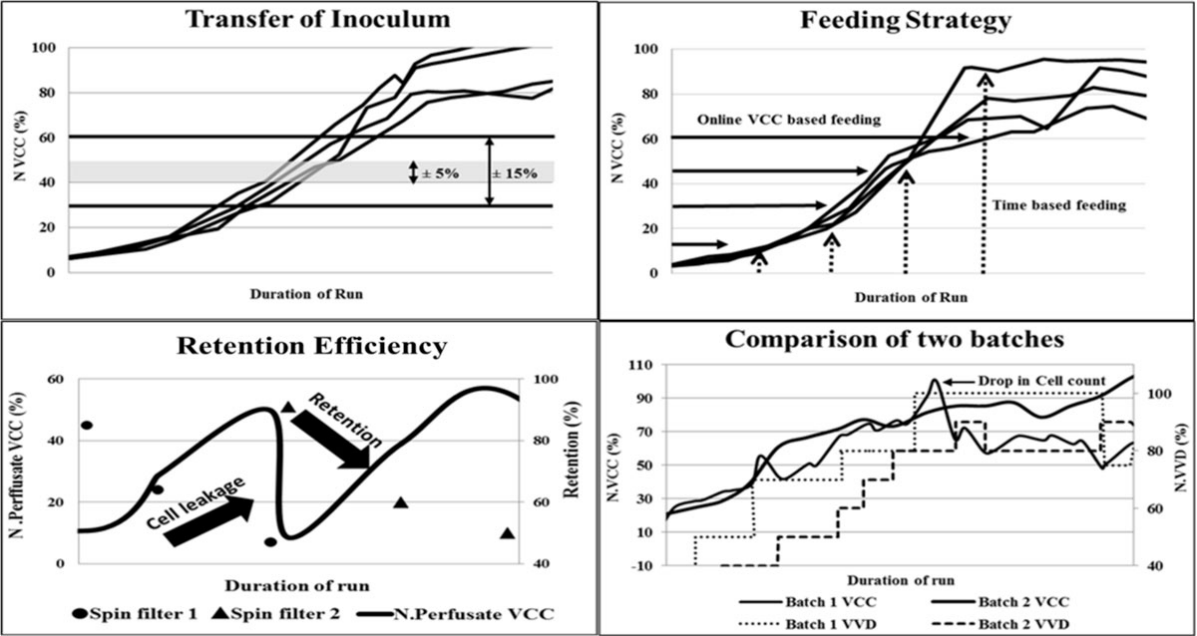
**(In clockwise direction) **a) Inoculum transfer range using offline (±15%) and online (±5%) VCC measurements. **b) **Feeding strategy comparison based on time and online probe readings **c) **Profiles of batches comparing N. VCC (Normalized VCC) and N. VVD. **d) **Drop in cell retention leading to increased cell leakage through the filter.

By measuring VCC online using a biomass probe, it was possible to transfer the inoculum at much precise cell concentration thus achieving consistent volumetric inoculum ratios in production bioreactor (Figure 1a).This resulted in an improved consistency in the cell culture profiles of the production run.

#### Feeding based on online VCC measurements

A Fed Batch process requires frequent additions of nutrient feeds to the bioreactor. These feeds are generally added either by sampling and measuring concentrations of residual nutrients or based on predefined culture time intervals. By feeding based on fixed culture duration, nutrients are added at same age but at different cell concentration. Feeding based on biomass probe readings helped in maintaining the nutrients as per VCC, thus preventing accumulation or depletion of nutrients in the process and eliminating batch-to-batch variations (Figure 1b).

### Case 2: Improving process efficiency in Perfusion

In our process, loss in cell-retention in the perfusion device led to decrease in cell conc. and productivity. By monitoring retention continuously, corrective actions could be taken to reduce these losses. Introducing a biomass probe in the perfusate line overcame operational constraints of frequent sampling to monitor retention efficiency.

#### Effective switching of the retention filters

As the SF clogs, there is a drop in perfusate volume being drawn from the filter, which results in pressure drop in the harvest line. Whenever the line pressure drops significantly, the perfusion is switched to the other filter. Calculations show reduction in retention efficiency of the filters from about 90% to 50% (Figure 1c). This reduction indicates cell loss through the filter resulting in significant drop in bioreactor VCC (Figure 1d).

To prevent a significant loss of cells from the bioreactor, it was decided to switch the filter by monitoring the retention by biomass probe in the perfusate line. Two biomass probes were inserted in the bioreactor and the perfusion outlet to measure the bioreactor cell concentration and the cells lost through the filter during perfusion. The filter was switched when the retention efficiency drop below 70%. This helped in preventing significant loss of viable cells from the bioreactor due to cell leakage through filters.

#### Effective control of perfusion rates

The perfusion rate in a perfusion run is generally reported as VVD (volume of medium perfused per bioreactor volume per day). As the VCC in the bioreactor increases, VVD is increased to provide additional nutrients for the cells. Although increase in VVD favours higher cell concentration, a drop in bioreactor VCC is also seen occasionally at higher VVD (Figure 1d, batch 1). Upon investigation it was evident that in these cases when VVD was increased, cell concentration in the bioreactor decreased due to increased cell leakage through the filters. Hence it was decided to control the VVD based on cell retention values. The VVD in the batch 2 was gradually increased considering the retention efficiency of the filter. A higher VCC was obtained in this batch compared to batch 1 even at lower VVD, due to lower cell loss through the filters.

## Summary

In the current study, effective use of biomass probe was demonstrated in applications ranging from direct measurement of VCC to indirect applications during perfusion. The probe can be used for these and similar applications as an effective PAT tool to improve process consistency and robustness.

